# Protective and risk factors for women’s mental health after a spontaneous abortion*

**DOI:** 10.1590/1518-8345.3382.3350

**Published:** 2020-09-07

**Authors:** Francine deMontigny, Chantal Verdon, Sophie Meunier, Christine Gervais, Isabel Coté

**Affiliations:** 1 Université du Québec en Outaouais, Gatineau, Qc, Canada.; 2 Scholarship holder at the Canadian Research Chair in Psychosocial Family Health, Canada.; 3 Université du Québec à Montréal, Montreal, Qc, Canada.; 4 Scholarship holder at the Fonds Québécois de Recherche en Santé, Canada.

**Keywords:** Abortion, Spontaneous, Nursing, Women, Mental Health, Emigrants and Immigrants, Protective Factors, Aborto Espontâneo, Enfermagem, Mulheres, Saúde Mental, Emigrantes e Imigrantes, Fatores de Proteção, Aborto Espontáneo, Enfermería, Mujeres, Salud Mental, Emigrantes e Inmigrantes, Factores Protectores

## Abstract

**Objective:**

to examine personal and contextual protective and risk factors associated with women’s mental health after a spontaneous abortion.

**Method:**

a cross-sectional study was carried out where 231 women who had experienced spontaneous abortions in the past 4 years answered a self-reporting online questionnaire to assess their mental health (symptoms of depression, anxiety, perinatal grief) and to collect personal as well as contextual characteristics.

**Results:**

women who had experienced spontaneous abortions within the past 6 months had higher scores for depressive symptoms than those who had experienced spontaneous abortions between 7 and 12 months ago, while anxiety level and perinatal grief did not vary according to the time since the loss. Moreover, low socioeconomic status, immigrant status, and childlessness were associated with worse mental health after a spontaneous abortion. In contrast, the quality of the conjugal relationship and the level of satisfaction with health care were positively associated with women’s mental health.

**Conclusion:**

women in vulnerable situations, such as immigrants, women with a low socioeconomic status, or childless women are particularly vulnerable to mental health problems after a spontaneous abortion. However, beyond those personal and contextual factors, the quality of the conjugal relationship and the level of satisfaction with health care could be important protective factors.

## Abstract (pt)

**Objetivo:**

examinar os fatores pessoais e contextuais de proteção e de risco associados à saúde mental das mulheres após aborto espontâneo.

**Método:**

foi realizado um estudo transversal, no qual 231 mulheres que sofreram aborto espontâneo nos últimos quatro anos responderam a um questionário *on-line*, cujo intuito era avaliar a saúde mental (sintomas de depressão, ansiedade, luto perinatal) e coletar informações pessoais, além de características contextuais.

**Resultados:**

mulheres que sofreram aborto espontâneo nos últimos seis meses apresentaram escores mais altos para sintomas depressivos do que mulheres que sofreram aborto espontâneo entre sete e 12 meses atrás, ao passo que o nível de ansiedade e o luto perinatal não variaram de acordo com o tempo transcorrido desde a perda. Além disso, baixo nível socioeconômico, status de imigrante e ausência de filhos foram associados a pior saúde mental após aborto espontâneo. Por outro lado, a qualidade do relacionamento conjugal e a satisfação com a assistência à saúde foram associadas positivamente à saúde mental das mulheres.

**Conclusão:**

mulheres em situação de vulnerabilidade, como as imigrantes, com baixo nível socioeconômico ou sem filhos estão particularmente vulneráveis a problemas de saúde mental após um aborto espontâneo. No entanto, além desses fatores pessoais e contextuais, a qualidade do relacionamento conjugal e a satisfação com a assistência à saúde podem ser importantes fatores de proteção.

## Abstract (es)

**Objetivo:**

examinar factores de protección y de riesgo personales y contextuales asociados a la salud mental de la mujer después de un aborto espontáneo.

**Método:**

se llevó a cabo un estudio transversal en el que 231 mujeres que habían sufrido un aborto espontáneo en los últimos 4 años respondieron a un cuestionario *online* de autoinforme para evaluar su salud mental (síntomas de depresión, ansiedad, duelo perinatal) y para recopilar características personales y contextuales.

**Resultados:**

las mujeres que habían sufrido un aborto espontáneo en los últimos 6 meses obtuvieron una puntuación más alta en lo que respecta a síntomas de depresión que las que lo habían sufrido entre 7 y 12 meses atrás, mientras que el nivel de ansiedad y el duelo perinatal no variaron según el tiempo transcurrido desde la pérdida. Además, la baja condición socioeconómica, el estado de inmigración y la falta de hijos se asociaron con una peor salud mental después de un aborto espontáneo. En cambio, la calidad de la relación conyugal y la satisfacción con la atención de la salud se asociaron positivamente con la salud mental de las mujeres.

**Conclusión:**

las mujeres en situaciones vulnerables, como las inmigrantes, las de baja condición socioeconómica o las mujeres sin hijos son especialmente vulnerables a problemas de salud mental después de un aborto espontáneo. Sin embargo, más allá de esos factores personales y contextuales, la calidad de la relación conyugal y la satisfacción con el cuidado de la salud podrían ser importantes factores de protección.

## Introduction

In Western societies, it has been estimated that approximately 20% of the pregnancies end in spontaneous abortions (also known as miscarriages) within the first 22 weeks^(^ 1 ^-^ 2 ^)^. The exact numbers are not known, as most countries only collect statistics for later perinatal deaths, that is, after the 24^th^ gestational week or when the foetus weighs more than one pound^(^ 3 ^)^. Despite this high prevalence, bereavement associated with spontaneous abortions has received much less attention from the scientific and professional communities than that associated with any other type of death^(^ 4 ^-^ 5 ^)^. The repercussions of bereavement related to early perinatal loss are nevertheless a significant issue for society, particularly in terms of public health, since a number of studies have indicated its important deleterious effects on women’s mental health^(^ 6 ^-^ 8 ^)^. Those findings raise an important question about protective and risk factors that may exacerbate or diminish the negative effects of spontaneous abortions on women’s mental health^(^ 9 ^)^. Answers to this question are crucial in order to better target women who are more at risk of developing mental health problems after a spontaneous abortion and to offer appropriate support. 

There are personal and contextual variables influencing women’s mental health after a miscarriage. A few authors have examined different personal and contextual variables that may influence women’s mental health after a spontaneous abortion and which act as protective or risk factors. Regarding the personal variables, some findings indicated that sociodemographic factors (e.g., age, socioeconomic status) were not associated with women’s mental health after a spontaneous abortion^(^ 3 ^,^ 6 ^)^. However, the majority of the studies on the experience of spontaneous abortion were conducted among white middle-class women living in a marital relationship^(^ 3 ^)^. Women living in conditions of vulnerability (low income, less schooling, or immigrant status)^(^ 3 ^)^ have received less research attention; as such, there is a need to assess the influence of these variables among more heterogeneous samples. In this vein, a study carried out with a large sample of Australian women found that the schooling level was positively associated with mental health after a spontaneous abortion^(^ 10 ^)^. The contextual variables of childlessness, advanced gestational age, and spontaneous abortion history were identified as factors that intensify perinatal bereavement^(^ 6 ^,^ 11 ^)^. However, except for childlessness, the results concerning these variables are inconsistent. Indeed, some researchers found that advanced gestational age and prior loss were associated with worse mental health^(^ 11 ^-^ 13 ^)^, while others found no significant association^(^ 11 ^)^.

The support offered to the parents by family members and friends or by health care professionals is another variable of interest. The result of an earlier study indicates that support from their spouse was especially important in helping women cope with spontaneous abortions^(^ 14 ^)^. Thus, it may be that the quality of the conjugal relationship has more influence on women’s mental health after a spontaneous abortion than the simple fact of being in a couple. The level of satisfaction with health care may also be important to mitigate the deleterious effects of spontaneous abortions on women’s mental health. Indeed, health care professionals are key actors in the health pathway of couples living through the experience of a spontaneous abortion^(^ 4 ^,^ 15 ^)^. However, even though the experience and repercussions of bereavement have been studied, women’s experiences of professional interventions-or the absence thereof-have received minimal research attention. 

Findings from the few qualitative studies on the women’s experience of spontaneous abortions have indicated that they most often report being dissatisfied with the health care received before, during, and after their spontaneous abortions^(^ 4 ^-^ 5 ^)^. Women lament the lack of compassion^(^ 14 ^)^, of social and emotional support^(^ 16 ^-^ 17 ^)^, and of information provided by the health care professionals^(^ 4 ^-^ 5 ^,^ 14 ^,^ 16 ^)^. From a quantitative perspective, the level of satisfaction with the primary care physician^(^ 10 ^)^ has been associated with better mental health, highlighting the important role of these health professionals in the recovery from a spontaneous abortion. However, other health care professionals (e.g., nurse, social worker) who might be involved in the care of women who had experienced a spontaneous abortion were not considered in this study^(^ 10 ^)^. In summary, the complex psychological phenomenon of a spontaneous abortion remains poorly studied, except with regard to grief reactions. Moreover, few researchers have examined women’s mental health after a spontaneous abortion in relation to their personal and contextual characteristics and the results have been inconsistent.

The objective of this study was to examine personal and contextual protective and risk factors associated with women’s mental health after a spontaneous abortion. The authors aimed to investigate whether the mental health of women who experienced spontaneous abortions varied in relation to various personal risk factors related to their age, socioeconomic status (income and schooling), and immigrant status. The authors also examined the effect of contextual risk factors, such as childlessness, number of gestational weeks, number of spontaneous abortions, and time elapsed since the spontaneous abortion, on these women’s mental health. Finally, the authors examined the association between two potential protective factors, the quality of the conjugal relationship and the level of satisfaction with health care, and the mental health of women who have experienced a spontaneous abortion. 

## Method

This cross-sectional study was conducted in the province of Québec, Canada, where an estimated 20,000 miscarriages occur for 86,000 live births^(^ 18 ^)^. It was approved by the research ethics board of the *Université du Québec en Outaouais* #1799. Ads were posted in medical clinics, on social media and on various websites, inviting women who had experienced a spontaneous abortion within the past four years to participate in the study. To be eligible to participate, women had to: 1) be aged 18 years or older; 2) have experienced at least one spontaneous abortion in the past four years; and 3) be able to read French. There were no exclusion criteria. The eligible participants were invited to read and sign a consent form and to answer an online questionnaire on a secure web platform. A total of 231 women completed the self-reporting questionnaire, which consisted of a set of six measures assessing mental health, personal and contextual variables, the quality of the conjugal relationship, and the level of satisfaction with health care.

*Mental health:* Depressive symptoms were assessed using the French version of the Edinburgh Postnatal Depression Scale^(^ 19 ^-^ 20 ^)^. On a 4-point Likert scale, the participants indicated how they felt in the past 7 days (e.g., *I have felt sad or miserable*). The 10 items were summed up to form a global depression score (α = .88). A score of 10 or higher was used as a cut-off point for possible depression^(^ 19 ^)^. Anxiety was assessed using the 20 items of the state subscale of the French version of the State-Trait Anxiety Inventory (STAI-S)^(^ 21 ^-^ 22 ^)^. On a 4-point Likert scale, the participants indicated how they felt right now, at this moment (e.g., *I feel upset*). The reversed scores were recoded and the items were summed up to produce a global anxiety score (α = .94). Perinatal grief was measured using the French version of the Perinatal Grief Scale^(^ 23 ^-^ 24 ^)^. On a 5-point Likert scale, the participants indicated their agreement with 33statements related to the loss of their baby (e.g., *I get upset when I think about the baby*). The reversed scores were recoded and the items were summed up to produce a global perinatal grief score (α = .94). 

*Personal and contextual variables:* The participants indicated their highest level of schooling, their family income, and their immigrant status. The answers related to schooling were grouped into three categories based on the diplomas or degrees obtained: high school, college (technical/vocational or pre-university), and university. Family income was categorized as ≤ 49,999 Canadian dollar (CAD), 50,000-99,999 CAD, or ≥ 100,000 CAD. The immigrant status was categorized into two groups: Non-immigrant (born in Canada) and Immigrant (other country of origin). The participants also indicated the date of their last spontaneous abortion, the number of previous spontaneous abortions, their age at the time of the last spontaneous abortion, and the gestational age of that foetus. The two latter variables were used as continuous variables. The time since the spontaneous abortion was classified as within the past 6 months, between 7 and 12 months, between 1 and 2 years, and between 2 and 4 years, while the number of spontaneous abortions was classified as 1, 2, or 3 or more. Finally, the participants indicated whether they had any living children of whom they were biological parents (yes/no).

*Quality of the conjugal relationship*: The quality of the conjugal relationship was measured using 4 items of the French version of the Dyadic Adjustment Scale^(^ 25 ^-^ 26 ^)^. On a 6-point Likert scale, the participants indicated their overall degree of happiness in their relationship and how often specific situations relating to their relationship usually happened (e.g., *In general, how often do you think that things between you and your partner are going well?*). Internal consistency was satisfactory (α = .81).

*Satisfaction with health care:* The level of satisfaction with health care was assessed using the Institutional Support Evaluation Scale^(^ 27 ^)^. On a 4-point Likert scale, the participants indicated how helpful they perceived a list of 19 different health services received during the course of their spontaneous abortion (nurse, physician, gynecologist) or afterwards (support groups). The participants could also indicate “not applicable” if they had not received the service. The scores for each service received were averaged to obtain a global satisfaction score (α = .95).

**Figure 1:**
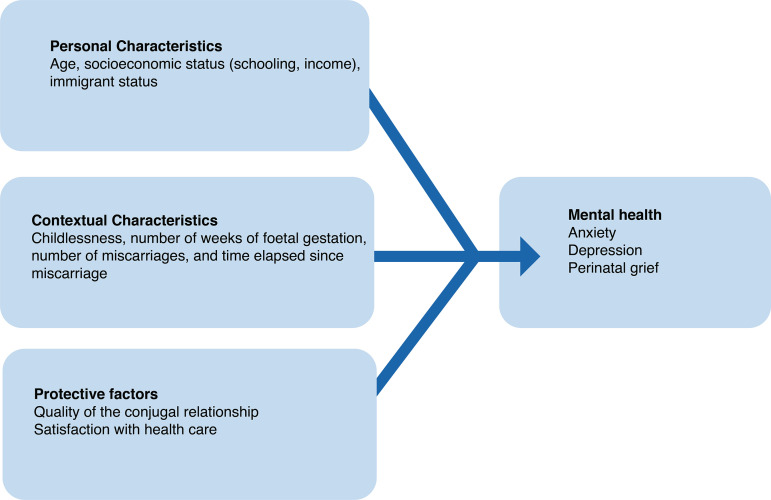
Variables related to the women’s mental health after a spontaneous abortion

The analyses were performed using SPSS v.22 (IBM). There were no missing variables for the measures of mental health and quality of conjugal relationship. For the level of satisfaction with health care, personal and contextual variables, there were few missing data *per* variable (from 1 to 7 *per* variable - less than 4%). Since these data were also randomly distributed, they were not imputed, and listwise deletion was used for main analysis^(^ 28 ^)^. Descriptive statistics (mean, standard deviation, proportion) was conducted to characterize the sample. One-way analyses of variance (ANOVAs) for the categorical variables, and correlations for the continuous variables, were then performed to examine associations between personal and contextual factors and women’s mental health. Tukey *post hoc* tests were performed when the ANOVAs indicated a significant difference and the independent variable comprised more than two groups (e.g., income, schooling). Hierarchical regression analyses were performed to examine the association between the quality of the conjugal relationship and the level of satisfaction with health care and mental health, while controlling for personal and contextual factors. In the first step, the contextual and personal variables that were significantly associated with mental health in the ANOVAs and the correlations were entered into the analysis. The quality of the conjugal relationship was entered in a second step, followed by the level of satisfaction with health care services in a third step. The statistical power analysis performed with G*power^(^ 29 ^)^ indicate that, to achieve a statistical power of 95%, with an average effect size, and 8 independent variables (quality of the conjugal relationship, level of satisfaction with health care, and up to six control variables), a sample of 160 participants is needed. Thus, the sample of the current study (n=231) provides a satisfactory statistical power. Figure 1 illustrates the study variables included in the analysis.

## Results

Table 1 presents descriptive statistics for the study variables. The women’s ages at the time of their last spontaneous abortion ranged from 19 to 43 years old, with a majority between 25 and 35 years old (n=170, 74%). A minority of the participants were immigrants or had a low socioeconomic status. For half of the women, the spontaneous abortion had happened less than 1 year ago, with the time since the spontaneous abortion ranging from 1 month to 4 years. Foetal gestational age at the time of the spontaneous abortion ranged from 3 to 20 weeks. The number of spontaneous abortions ranged from 1 to 11, with a majority of women having experienced 1 or 2 miscarriages. Nearly 40% of the participants were childless at the time of the study. The women were not asked if they were pregnant at the time of data collection, which could be a limitation of this study. Regarding age, income and schooling, the sample is representative of the women in the region^(^ 18 ^)^.

**Table 1:** Descriptive statistics for the study variables. Québec, Canada, 2018

Variables	N (%)
Family income (CAD)	
0-49,999	53 (23.14%)
50,000-99,999	100 (43.67%)
≥ 100,000	76 (33.19%)
Schooling level	
High school	34 (14.85%)
College	67 (29.26%)
University	125 (54.59%)
Immigrant status	
Immigrant	17 (7.42%)
Non-immigrant	212 (92.58%)
Time since spontaneous abortion	
0-6 months	64 (27.95%)
7-12 months	58 (25.33%)
1-2 years	64 (27.95%)
2-4 years	43 (18.78%)
Number of spontaneous abortions	
1	150 (65.50%)
2	34 (14.85%)
3 or more	45 (19.65%)
Childlessness	
Yes	87 (37.99%)
No	142 (62.01%)
	**Mean (SD)**
Age	30.74 (4.60)
Gestational age of the foetus	9.35 (3.35)
Depression (/30)	10.85 (6.40)
Anxiety (/80)	37.66 (11.89)
Perinatal grief (/165)	72.02 (29.02)
Quality of the conjugal relationship (/20)	15.81 (3.28)
Satisfaction with health care	3.11 (0.63)

Regarding the mental health variables, 125 (55%) women had a depression score greater than or equal to 10, indicating possible depression^(^ 19 ^)^, and 62 (27.1%) reported high scores of perinatal grief (>91)^(^ 30 ^)^. The anxiety mean score was moderate, and 42 (18.3%) participants reported low-quality conjugal relationship (score <14)^(^ 25 ^)^. Finally, on average, the participants felt relatively satisfied with the health care services (mean score of 3.11/4.00). 

Table 2 presents the results of the one-way ANOVA testing differences in the mental health scores according to personal and contextual characteristics. The results first indicated that women’s mental health varied in line with their income and their schooling level. In fact, *post hoc* tests revealed that women with a family income ≤ 49,999 CAD had higher scores for depression, anxiety, and perinatal grief than those with a family income ≥ 50,000 CAD, while women with a high school diploma had higher scores for perinatal grief than those with a college or university degree. Immigrant women also had higher scores for depression and perinatal grief than non-immigrant women, while childless women reported significantly more depression, anxiety, and perinatal grief symptoms. The women’s depression levels differed according to the time since the spontaneous abortion. Indeed, the *post hoc* tests indicated that women who had miscarried within the past 6 months had higher scores for depression than those who had miscarried between 7 and 12 months ago. There were no significant differences in the mental health scores according to the number of spontaneous abortions.

**Table 2:** Women's mental health according to personal and contextual variables. Québec, Canada, 2018

Variables	N	Depression Mean (SD)	F	*p*	Anxiety Mean (SD)	F	*p*	Perinatal grief Mean (SD)	F	*p*
Income (CAD)										
0-49,999	53	14.02 (5.74)	9.14	0.000	42.02 (11.92)	4.83	0.010	90.32 (29.85)	15.58	0.000
50,000-99,999	100	10.07 (6.84)			36.60 (13.26)			67.34 (28.27)		
≥ 100,000	76	9.67 (5.53)			36.03 (9.03)			65.41 (24.01)		
Schooling										
High school	34	12.59 (6.19)	1.70	0.128	40.15 (12.90)	1.22	0.239	88.82 (29.24)	10.02	0.000
College	67	10.39 (6.82)			37.72 (12.23)			73.99 (31.82)		
University	125	10.44 (6.08)			36.62 (11.17)			65.40 (24.57)		
Immigration status										
No*	212	10.55 (6.32)	6.41	0.013	37.27 (11.71)	3.18	0.080	70.26 (28.11)	10.88	0.001
Yes^†^	17	14.59 (6.42)			42.59 (13.39)			93.88 (32.07)		
Time since spontaneous abortion										
0-6 months	64	12.61 (5.71)	2.85	0.046	40.25 (12.27)	1.50	0.209	78.63 (26.78)	2.45	0.095
7-12 months	58	9.66 (6.51)			36.12 (21.14)			68.88 (30.22)		
1-2 years	64	10.97 (6.37)			37.23 (11.70)			73.45 (31.90)		
> 2 years	43	9.67 (6.83)			36.53 (11.01)			64.28 (24.16)		
Number of spontaneous abortions										
1	150	10.86 (5.77)	0.24	0.785	37.10 (11.47)	1.04	0.381	69.25 (26.81)	2.86	0.068
2	34	10.26 (7.28)			37.12 (12.08)			72.38 (30.49)		
≥ 3	45	11.27 (7.69)			39.96 (13.07)			80.96 (33.52)		
Childlessness										
No	142	10.08 (6.02)	5.58	0.013	35.40 (10.69)	14.32	0.000	64.93 (24.66)	24.61	0.000
Yes	87	12.11 (6.81)			41.36 (12.85)			83.59 (31.88)		

* Non-immigrant;

† Immigrant

The correlations analysis (Table 3) indicated that neither the age at the time of the spontaneous abortion nor the gestational age of the foetus were significantly associated with depression, anxiety, or perinatal grief. However, the quality of the conjugal relationship and the level of satisfaction with health care were significantly positively correlated with those three mental health variables. Depression, anxiety, and perinatal grief were also strongly positively correlated.

**Table 3:** Correlations among the study variables (continuous variables). Québec, Canada, 2018

Variables	Gestational age of the foetus	Age at spontaneous abortion	Quality of the conjugal relationship	Satisfaction with health care	Depression	Anxiety	Perinatal grief
Gestational age of the foetus							
Age at spontaneous abortion	.199^†^						
Quality of the conjugal relationship	-.067	-.217^†^					
Satisfaction with health care	.053	-.015	.088				
Depression	.085	.008	-.245^†^	-.287^†^			
Anxiety	.042	.054	-.292^†^	-.169*	.727^†^		
Perinatal grief	.070	.041	-.288^†^	-.265^†^	.689^†^	.675^†^	

* p<.05,

† p<.01

Finally, linear hierarchical regressions were performed to examine the association between the quality of the conjugal relationship, the level of satisfaction with health care, and mental health, while controlling for personal and contextual variables. Since income, schooling level, immigration status, time since spontaneous abortion, and childlessness were significantly associated with depression, anxiety, and perinatal grief, they were included as covariates in the first step of the analysis. To include schooling level, income, and time since spontaneous abortion in the regression analysis, dummy variables were created (income: 0 = ≤ 49,999, 1 = ≥ 50,000; schooling level: 0 = high school, 1 = college or university; time since spontaneous abortion: 0 = ≤ 6 months; 1 = > 6 months). The results, presented in Table 4, indicated that income was negatively associated with depression, anxiety and perinatal grief, while time since spontaneous abortion was negatively associated with depression and perinatal grief. Schooling was also negatively associated with perinatal grief, while childlessness was positively associated with anxiety and perinatal grief. The results also indicated that, even after controlling for those personal and contextual variables, the quality of the conjugal relationship explained a significant amount of variance in depression, anxiety, and perinatal grief and was significantly negatively associated with those three variables. Finally, even after controlling for all the variables mentioned above, the level of satisfaction with health care added to the prediction of depression and perinatal grief and was significantly and negatively associated with those two variables.

**Table 4:** Linear hierarchical regression analysis for the association between quality of the conjugal relationship, satisfaction with health care and women's mental health, controlling for personal and contextual variables. Québec, Canada, 2018

Variables	Β	*p*	Depression		Β	*p*	Anxiety		Β	*p*	Perinatal grief	
			95% Confidence Interval				95% Confidence Interval				95% Confidence Interval	
			Lower	Upper			Lower	Upper			Lower	Upper
**Step 1**												
Income (≥ 50,000 CAD)	-.20	.004	-4.97	-0.92	-.14	.051	-7.53	0.02	-.21	.001	-22.47	-5.66
Schooling (college or university)	-.04	.512	-3.09	1.54	-.04	.583	-5.54	3.12	-.19	.003	-24.27	-4.99
Immigrant status	.07	.319	-1.62	4.96	.03	.632	-4.66	7.65	.10	.121	-2.87	24.50
Time since spontaneous abortion (> 6 months)	-.17	.010	-4.06	-0.55	-.11	.088	-6.15	0.43	-.12	.048	-14.68	-0.06
Childlessness	.10	.130	-0.38	2.93	.22	.001	2.09	8.27	.25	.000	7.53	21.28
R^2^	.10				.09				.21			
**Step 2**												
Quality of the conjugal relationship	-.27	.000	-0.74	-0.26	-.31	.000	-1.54	-0.65	-.31	.000	-3.57	-1.60
R^2^	.17				.18				.30			
**Step 3**												
Satisfaction with health care	-.22	.001	-3.32	-0.91	-.09	.153	-3.93	0.62	-.16	.007	-11.86	-1.94
R^2^	.21				.19				.32			

## Discussion

The main objective of the present study was to identify personal and contextual variables that can represent risk factors for women’s mental health after a spontaneous abortion. Specific attention was also given to two potential protective factors: quality of the conjugal relationship and satisfaction with health care. The results showed a high rate of depression among the women in the study. Indeed, half of them could be classified as possibly depressed. This proportion is much higher than the rate of postpartum depression among Canadian women overall (8.69%)^(^ 31 ^)^. 

The results also indicated that the women who had miscarried within the past 6 months had higher scores for depression than those who had miscarried 7 to 12 months ago. Those results are consistent with previous studies indicating that most women recover from spontaneous abortion within approximately 6 months^(^ 32 ^)^. However, in the present study, the anxiety level and perinatal grief did not vary according to the time since the spontaneous abortion. These results suggest that, for some women, the symptoms persist long after the loss. In fact, there may be several reasons why symptoms of psychological distress and mental health would fluctuate over time, including difficulties conceiving another child, and anniversaries or other significant dates associated with the spontaneous abortion. 

Along these lines, a previous study^(^ 12 ^)^ had found that the association between prenatal loss and depression and anxiety did not differ significantly over time and could persist for up to 3 years. A recent study^(^ 6 ^)^ indicated that depressive and grief symptoms persist longer for women who are childless or dissatisfied with the health care services. Future studies should continue on this path and investigate other moderating variables, such as number of spontaneous abortion, age, infertility history, and previous other losses, which might shed light on the conditions under which depression, perinatal grief, and anxiety are likely to resorb more quickly after a spontaneous abortion.

Concerning personal and contextual characteristics, our results suggest that immigrant women, as well as those with a low socioeconomic status and childless women, are particularly vulnerable to mental health problems after a spontaneous abortion. These results are consistent with a previous comprehensive review^(^ 33 ^)^, which identified childlessness as an important contextual variable influencing women’s mental health after a spontaneous abortion. However, our results contradict the finding of that review which indicates that the sociodemographic variables were not associated with mental health after a spontaneous abortion. These non-significant results may be due to the fact that past studies used homogenous samples composed of white middle-class married women. Although our sample contained only a small number of immigrants and women with a low socioeconomic status, we were able to identify those two variables as significantly influencing women’s mental health. 

The results of our study are consistent with a previous study^(^ 34 ^)^ which had found a positive association between schooling level and mental health among women after a spontaneous abortion. However, because no control group was used in the present study, it was not possible to isolate the effect of the spontaneous abortion on those women’s mental health in relation to their immigration or socioeconomic status. Also, no data was collected about the mental health of those women before their spontaneous abortions. Nevertheless, it is conceivable that their spontaneous abortions added to other stressors already associated with those personal characteristics^(^ 35 ^)^. As those women may have fewer resources to cope with life stressors^(^ 36 ^)^, they constitute a vulnerable population particularly at risk of developing mental health problems after a spontaneous abortion, and it is clearly necessary to understand their needs and to provide relevant services for them. To clarify those associations, future studies would need to be undertaken with a larger number of immigrant women and women with a low socioeconomic status, along with a control group of women with those same characteristics who did not miscarry.

Women’s mental health did not differ according to the number of spontaneous abortions. The results concerning the association between spontaneous abortion history and mental health have been inconsistent^(^ 3 ^)^. The moderating variables could probably explain under which circumstances the number of spontaneous abortions might influence mental health. For example, having experienced a previous pregnancy loss (including a spontaneous abortion) has been associated with poorer mental health during a subsequent pregnancy, but not in the postpartum period following the birth of a live child^(^ 37 ^)^. Thus, the presence of another child seems to moderate the association between a prior loss and mental health. 

The results of the linear hierarchical regression indicated that, even after controlling for socioeconomic status, immigrant status and childlessness, the quality of the conjugal relationship explained a significant amount of the variance in depression, anxiety, and perinatal grief, and was negatively associated with those three variables. Thus, women who had miscarried and were satisfied with their relationship experienced better mental health. However, this association could also be bidirectional, such that mental health could in turn influence the quality of the conjugal relationship. Indeed, in addition to these repercussions on women’s mental health, spontaneous abortions can also impact on the conjugal relationship. 

A number of studies have observed a diminished level of satisfaction with the relationship following a perinatal death, which can even ultimately lead to separation or divorce^(^ 38 ^-^ 39 ^)^. The fear of another loss, the mothers’ anxiety, and differences in the two spouses’ reactions are elements that have been identified^(^ 38 ^)^ as contributing to conjugal difficulties^(^ 40 ^)^. Unfortunately, the cross-sectional nature of the present study prevented us from testing this hypothesis. Future studies using a longitudinal design could examine the bidirectional nature of the relation between the quality of the conjugal relationship and mental health, and attempt to determine which one is more likely to predict the other. Previous studies have indicated that people who were dissatisfied with their marriages, compared to those who were satisfied with their relationships, were 2.68 times more likely to develop an MDE^(^ 41 ^)^(Major Depressive Episode), while our sample was 3.34 times more at risk.

Moreover, even after controlling for sociodemographic variables, childlessness, and quality of the conjugal relationship, the level of satisfaction with health care added to the prediction of depression and perinatal grief. Since many women reported low levels of satisfaction with the health care services after their spontaneous abortion^(^ 5 ^-^ 6 ^,^ 17 ^,^ 42 ^)^, these results highlight the importance of paying particular attention to the quality of the services provided to women before, during, and after a spontaneous abortion. Qualitative studies examining women’s experience of a spontaneous abortion have noted that compassion and empathy from health professionals, provision of accurate information (e.g., cause of the loss, physical and emotional symptoms), involvement in treatment decisions, and social support from nurses were identified as particularly helpful^(^ 42 ^)^. These recommendations should be heeded when developing health care services for women who have miscarried.

Finally, our results indicated that the three mental health variables were strongly correlated with each other, indicating a high risk of comorbidity among women having experienced spontaneous abortions. However, personal and contextual variables seemed to be differently associated with those three variables. For example, in the hierarchical regression analysis, childlessness was most strongly associated with perinatal grief than with depression and anxiety when controlling for other sociodemographic factors. This highlights the importance of examining various mental health indicators when studying spontaneous abortions. As noted previously^(^ 34 ^)^, the fact that some women may not present anxiety and depression symptoms after a spontaneous abortion does not mean that they are not struggling with difficult emotions and trying to find meaning in their experience.

One limitation of the present study is its cross-sectional design, which does not allow us to comment on the direction of the association between the study variables. For example, it is possible that women with poorer mental health assessed the health care services they received more negatively. Also, since no control group was used, it was not possible to isolate the influence of spontaneous abortions from other risk factors for women’s mental health, and the results of the present study should be interpreted cautiously. Nevertheless, the results indicate worrying scores of depression, anxiety and perinatal grief among this sample of women who had experienced a spontaneous abortion in the last 4 years. Finally, the women’s histories of prior mental health problems, identified as factors that intensify perinatal bereavement^(^ 6 ^,^ 11 ^)^, and the use of medication or psychotherapy for depression or anxiety were not measured in the present study and might have explained part of the variance in depression, anxiety, and perinatal grief.

Nurses should pay special attention to immigrant women after a spontaneous abortion, in terms of monitoring for mental health issues (e.g., depression, grief, anxiety), and even more so if they have no other children or are living in precarious economic circumstances. Policy makers need to address these issues-for example, by proposing and supporting the implementation of practice guidelines in healthcare services.

## Conclusion

The present study adds to the sparse literature focusing on protective and risk factors for women’s mental health after a spontaneous abortion. The results indicate that the symptoms of anxiety, depression, and perinatal grief can persist for a long period after the loss. Also, more attention should be given to women in vulnerable situations, such as immigrant women, women with a low socioeconomic status, or childless women. Beyond those personal and contextual factors, the quality of the conjugal relationship and the level of satisfaction with health care appear to be important protective factors against mental health problems after a spontaneous abortion.
